# Self-care strategies and interventions to address antimicrobial resistance: evidence review and recommendations for national programs, service providers, caregivers, and self-carers

**DOI:** 10.3389/fpubh.2026.1782858

**Published:** 2026-09-03

**Authors:** Reuben Kiggundu, J. P. Waswa, Fahad Lwigale, Herman Mwanja, Mackline Hope, Conrad Tumwine, Rodgers Rodriguez Ayebare, Andrew Kambugu, Dathan M. Byonanebye, Francis Kakooza

**Affiliations:** 1 Department of Global Health Security, Infectious Diseases Institute, College of Health Sciences, Makerere University, Kampala, Uganda; 2 Department of Community Health and Behavioral Sciences, School of Public Health, Makerere University, Kampala, Uganda

**Keywords:** antimicrobial resistance, digital health, self-awareness, self-care, self-management, self-monitoring, self-testing and diagnosis, self-treatment

## Abstract

**Background:**

Antimicrobial resistance (AMR) poses a significant global health threat, disproportionately affecting low- and middle-income countries where fragile health systems struggle to respond effectively. Self-care strategies and interventions present a complementary approach to support antimicrobial stewardship by empowering individuals to prevent and manage infections with appropriate tools and guidance.

**Objective and key concepts:**

This narrative review provides an interpretive synthesis of the role of self-care strategies and interventions in AMR containment, focusing on five core self-care strategies: self-awareness through AMR education, self-testing using rapid diagnostics, guided self-treatment with quality-assured medicines, self-monitoring via digital tools, and structured self-management under professional oversight. Digital health and community health systems are self-care interventions that enable self-care strategies.

**Findings:**

Self-care strategies and interventions enhance early infection detection and management, promote appropriate antimicrobial use, and reduce pressure on health systems. However, effective implementation is hindered by limited access to reliable AMR information, risks of misdiagnosis and antibiotic misuse, privacy concerns, and behavioral challenges.

**Conclusion and recommendations:**

Integrating self-care into national programs is essential. Policymakers should prioritize policy integration, standardized guidance, regulation, infrastructure development, and research on self-care strategies and interventions. Self-care support programs should translate these priorities into community-level interventions through education, capacity building, and access initiatives. Telehealth providers can facilitate the safe use of digital tools and remote support, caregivers should promote correct practices and adherence, and self-carers should engage in informed self-care and provide feedback.

## 1 Introduction

Antimicrobial resistance (AMR) is a major global health challenge, directly causing an estimated 1.27 million deaths and contributing to nearly 5 million deaths globally (1). The burden is greatest in low- and middle-income countries (LMICs) where health systems are overburdened. Among children under five, the disparity is starker, with 99.65% of AMR-related deaths in this age group occurring in LMICs (2). Beyond mortality, AMR reduces productivity through prolonged illness (3). These losses extend to caregivers and healthcare workers, who must devote additional time and resources to managing resistant infections. To this end, its estimated that AMR could reduce global Gross Domestic Product (GDP) by up to 3.8% by 2050, with LMICs expected to bear the heaviest losses (4).

Antimicrobial resistance emerges from interlinked drivers across the human, animal, and environmental health sectors, making it a quintessential One Health challenge (5). In animal health, routine antibiotic use for growth promotion and disease prevention fosters cross-resistance and contamination of the food chain (6). In the environmental sector, inadequate wastewater treatment, pharmaceutical effluents, and agricultural runoff disseminate resistant organisms and antimicrobial residues into water and soil ecosystems (7). In the human health sector, antibiotic overuse and misuse, gaps in infection prevention and control (IPC), and limited diagnostic capacity contribute to the emergence and spread of AMR (8). The interaction of these factors amplifies AMR transmission and directly undermines containment efforts across all sectors. In the human health sector, AMR containment efforts are shaped by factors such as healthcare access, IPC measures, public awareness, and health system capacity (9). In LMICs, financial barriers, inadequate health facilities, and workforce shortages drive self-medication and inappropriate antimicrobial use (AMU), accelerating resistance (8, 10). Poor infection control in health facilities, stemming from overcrowding, poor sanitation, and inadequate hygiene, further accelerates the spread and emergence of resistant pathogens (11–13). Meantime, limited AMR awareness (14, 15) and regulatory gaps (16, 17) contribute to widespread inappropriate AMU in facilities (18, 19) and communities (20, 21). Additionally, underdeveloped surveillance systems in many LMICs hinder the ability to monitor resistance and inform effective national response efforts (22).

Health systems in LMICs are already overburdened, making it difficult to meet the growing demand for care and to effectively respond to the growing AMR challenge. Addressing AMR therefore requires not only traditional health system investments but also innovative, non-traditional approaches that reflect how people actually manage illness. One such approach is self-care, defined by the World Health Organisation (WHO) as the ability of individuals, families, and communities to maintain health, prevent disease, and manage illness, with or without support from healthcare professionals (HCPs) (23). Self-care is particularly important for addressing AMR, as it provides a practical avenue to promote appropriate AMU within communities (24). However, despite its potential, the role of self-care in AMR containment remains largely unexplored. Understanding how self-care practices influence AMU, identifying effective interventions, and integrating these approaches into broader AMR strategies is critical to closing existing knowledge gaps and unlocking community-level solutions that complement formal health system efforts. Self-care can be supported through interventions such as evidence-based medicines, self-diagnostic tools, digital health applications, wearable medical devices, and other devices. These can be delivered with or without direct healthcare supervision and are recognised by the WHO as essential strategies for achieving universal health coverage (25). When implemented safely and equitably, self-care interventions can ease the burden on overstretched health systems, increase individual autonomy, and expand access to timely, appropriate care (23). In the context of AMR, self-care is particularly important as it offers a practical means to improve AMU within communities (24).

As self-care is rapidly gaining prominence in LMICs (26). Ensuring safe, effective, and equitable access to self-care interventions is critical to effective AMR containment, requiring investments in education, supportive policy frameworks, and digital health technologies that enable informed decision-making. This paper explores self-care strategies and interventions, examines their role in AMR containment in limited-resource settings, synthesizes available evidence on their implementation and challenges, and offers actionable recommendations for national programs, service providers, caregivers, and self-carers.

## 2 Methods

The paper adopts a narrative, non-systematic review methodology to provide an interpretive, and policy-informing synthesis of literature on self-care strategies and interventions for AMR containment. The objective was not to systematically identify or quantitatively synthesize all available evidence, but rather to integrate current knowledge, conceptual perspectives, implementation experiences, and policy guidance to develop a coherent framework and evidence-informed recommendations. Accordingly, this review did not follow a predefined review protocol, undertake an exhaustive literature search, perform formal study screening, conduct standardized data extraction, assess risk of bias, or adhere to PRISMA reporting requirements, as these are methodological features of systematic reviews rather than narrative reviews.

Literature was identified through purposive searches of peer-reviewed publications, international guidelines, policy documents, and relevant grey literature using combinations of concepts related to AMR, infection prevention and management, antimicrobial stewardship, antibiotic use, self-care strategies and interventions, digital health, and community health. Sources were selected based on their conceptual relevance, methodological rigor, policy significance, and contribution to understanding self-care strategies and interventions for AMR containment, with particular emphasis on evidence from low- and middle-income countries. Additional key publications were identified through citation tracking of relevant articles and international guidance documents where appropriate.

Evidence was synthesized using a thematic analytical approach organized around the conceptual foundations of self-care, the five core self-care strategies, enabling interventions, implementation challenges, and multi-level recommendations. Rather than statistically combining findings, the review integrates empirical evidence with conceptual and policy frameworks to develop an interpretive understanding of how self-care can contribute to AMR containment and to identify priorities for policy, practice, and future research. As part of this interpretive synthesis, the evidence underpinning each self-care strategy and intervention was critically appraised to enhance transparency regarding the strength and applicability of the available evidence. Rather than undertaking a formal risk-of-bias assessment, we distinguished between interventions supported by direct evidence from AMR, antimicrobial use, antimicrobial stewardship, or infection prevention studies and those supported by indirect evidence from analogous health domains (e.g., HIV, tuberculosis, malaria, and chronic disease management), where similar self-care approaches have demonstrated effectiveness. This appraisal was informed by critical interpretation of the literature, biological plausibility, similarity of implementation mechanisms, and consistency of findings across related health conditions.

## 3 Conceptual basis for self-care for AMR containment

### 3.1 Foundations of self-care

Self-care is an integral part of every individual and society, intrinsically linked to survival and well-being, and has existed in some form since the dawn of humanity. Globally, self-care is often driven by challenges in accessing healthcare services, such as costs, distance, and workforce shortages (27). In LMICs, these self-care drivers are compounded by under-resourced health systems, long wait times, and limited availability of essential medicines and diagnostics (28). The recent COVID-19 pandemic highlighted the importance of community-led approaches to strengthen resilience, as traditional health facility-based systems alone were not designed or equipped to absorb such large-scale, sustained demand for services (29, 30). At the same time, the expanding internet connectivity and proliferation of social media have significantly improved health literacy and awareness (31, 32). This has accelerated the adoption of self-care interventions, particularly those leveraging technological advancements and digital connectivity (33).

**Figure 1:**
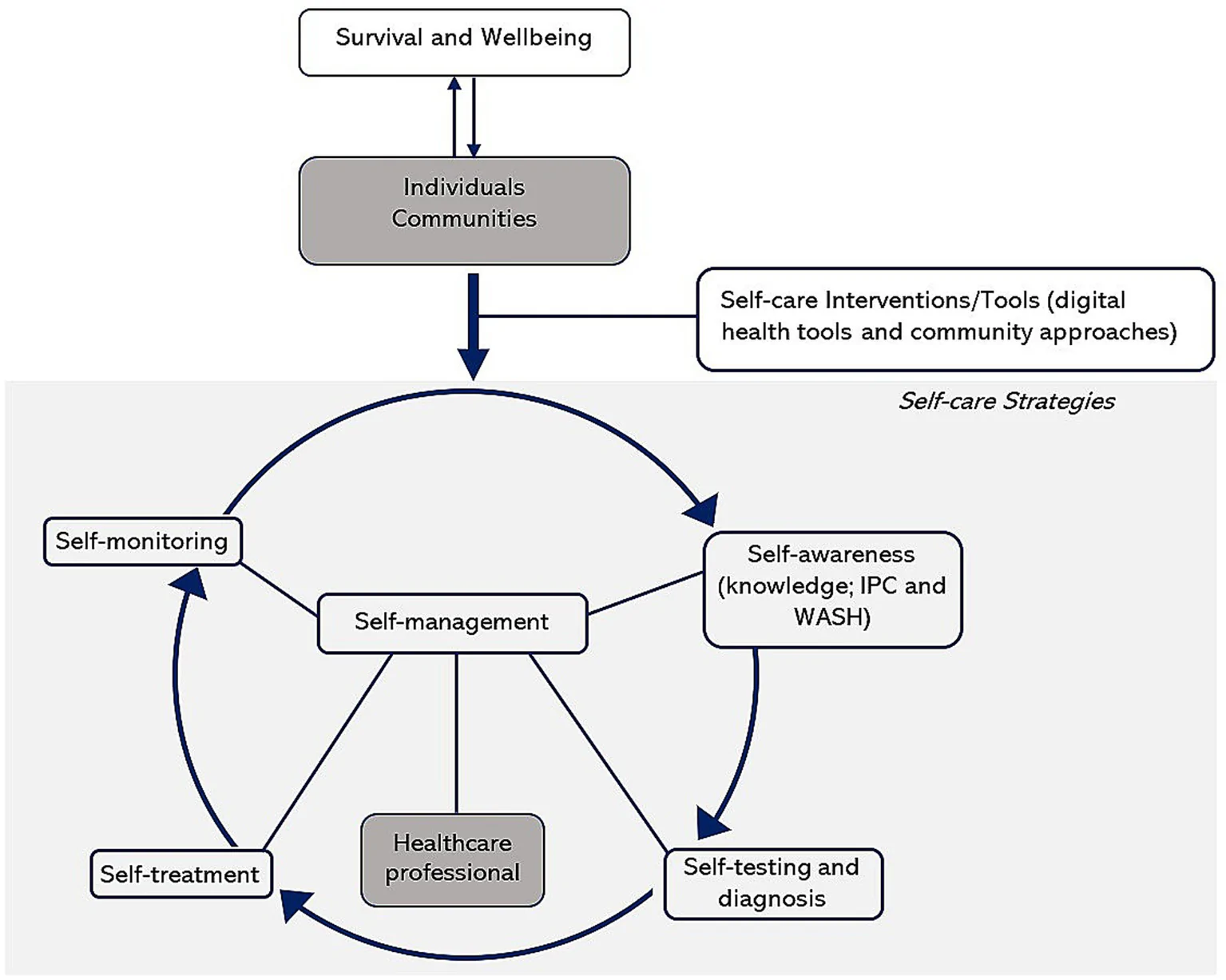
Foundations of self-care.

The fundamental human drive for survival and wellbeing, serving as an intrinsic mechanism, empowers individuals and communities to navigate health challenges in contexts where access to professional care is limited. Figure 1 illustrates the foundations of self-care. At its core, self-care involves individuals making informed health decisions and leveraging interventions to implement strategies that promote appropriate AMU, with or without oversight from HCPs. Self-care interventions are tools used by individuals, families, and communities, also known as self-carers, that utilize evidence-based, quality-assured medicines, devices, diagnostics, and digital tools with or without the support of HCPs, to manage health and promote well-being. These tools are applied through interconnected strategies, including self-awareness, self-testing and diagnosis, self-treatment, self-management, and self-monitoring (30).

The interconnection among these strategies is critical for ensuring optimal AMU and effective AMR containment. Self-awareness empowers individuals to recognize symptoms and understand AMR risks. This awareness can lead to informed decisions about self-testing and diagnosis, which in turn inform appropriate self-treatment and management choices that impact AMU (34). Additionally, self-monitoring practices enable individuals to track their health status and adjust their care accordingly, promoting adherence to treatments and preventing antibiotic misuse (35, 36), closing the loop back to heightened awareness. Central to this cycle is self-management, which integrates the other self-care strategies but becomes distinct when professional oversight is involved. In this context, the other strategies effectively morph into self-management, guided and refined through direct linkage with HCPs. This ensures coordinated escalation and decision-making, rather than isolated or fragmented flows between strategies. Collectively, these interconnected strategies are anchored in empowerment, equity, and evidence, and guided by individual autonomy and supportive policy (27). Tools should be designed to fit within this framework, providing individuals with ethical, safe, and effective options that are culturally appropriate, context-specific, and accessible to all (23).

### 3.2 Justification and rationale for self-care

In LMICs, the high burden of infectious diseases, compounded by constrained access to timely diagnostics and care, creates a context in which self-care practices are both necessary and inherently high-risk. Within this setting, AMR further disrupts the effectiveness of treatment and amplifies morbidity and mortality across individuals and communities embedded within interconnected human, animal, and environmental systems (37). From a One Health perspective, self-care cannot be considered in isolation from upstream infection dynamics, including household hygiene practices, water, sanitation and hygiene (WASH), infection prevention and control behaviors, antimicrobial use in animals, food safety systems, and environmental contamination, all of which shape both exposure risk and antimicrobial demand. These interactions directly influence self-care strategies and decisions such as perceived need for antibiotics, self-treatment and monitoring. However, they also introduce downstream risks, including delayed care-seeking, inappropriate antimicrobial use, reinfection, and onward transmission within households and communities, which may ultimately undermine the effectiveness of self-care strategies. Accordingly, infection prevention, situated within a broader One Health framework, forms a foundational paradigm underpinning self-care, as it directly shapes both the occurrence of infections and the demand for self-initiated care responses (38). Strengthening preventive measures across household, community, animal, and environmental interfaces is therefore not only critical for reducing infection incidence but also central to improving the safety, appropriateness, and effectiveness of self-care strategies in AMR containment (39).

The COVID-19 pandemic and similar infectious disease outbreak shocks have demonstrated that acute disruptions are often followed by prolonged periods of increased service demand, backlog in routine care, and opportunity costs for preventive and primary health services, thereby necessitating the reorganization of care pathways to include more decentralized and self-managed models of care (40). In addition, the growing burden of post-infectious sequelae and rehabilitation needs underscores how infectious disease emergencies extend well beyond the acute phase, generating sustained demand that can further constrain health system capacity and crowd out routine prevention and stewardship activities (41, 42). Within this context, appropriate and effective self-care becomes a pragmatic and necessary component of resilient health systems, particularly in the aftermath of the COVID-19 pandemic and recurrent infectious disease outbreaks in LMICs, which collectively intensify pressure on health services, disrupt routine care pathways, and are often associated with increased inappropriate antibiotic use and accelerated AMR (43, 44).

### 4. Self-Care Strategies and Interventions for AMR Containment

#### 4.1 Self-awareness

Self-awareness, as a self-care strategy, refers to an individual’s ability to recognize and understand their own health status, risk factors, and behaviors that influence wellbeing. It encompasses awareness of symptoms, bodily changes, infection risks, and preventive measures such as hygiene, nutrition, and healthy lifestyle practices (27). By fostering informed decisions about when to seek care or adopt self-care actions, self-awareness serves as the foundation upon which all other self-care strategies are built. Recognizing the existence of a problem and understanding its impact on oneself is the first step toward managing it effectively. In the context of AMR, self-awareness forms the foundation of effective self-care. It equips individuals with an understanding of infection basics (45), the causes and consequences of AMR, and the ways in which their behavior, lifestyle, and health practices influence health outcomes (46). This awareness fosters a sense of personal responsibility, motivating individuals to adopt protective behaviors, seek timely professional care, and use antimicrobials appropriately (47, 48). In doing so, they not only safeguard their own health but also contribute to the protection of their families and communities. By leading by example through good infection prevention practices, appropriate use of antimicrobials, and seeking and following professional guidance, individuals can play a role in AMR containment (49, 50).

##### 4.1.1 Self-awareness interventions for AMR containment

i *Public awareness campaigns and traditional community engagement*

Raising self-awareness is the critical first step in enabling individuals and communities to respond effectively to AMR. Global initiatives, such as the World Antimicrobial Resistance Awareness Week (WAAW), highlight the issue across the health workforce, policymakers, and the public (51); however, 1 week of heightened visibility per year is insufficient. Sustained, locally contextualized campaigns are needed to maintain public engagement and reinforce behavioral change (52). In many LMICs, where face-to-face health communication remains a major source of information, community-level approaches offer a culturally resonant and accessible way to promote AMR awareness. Storytelling, music, dance, and drama translate medical concepts into familiar, relatable terms, fostering participation, reflection, and dialogue around harmful practices such as self-medication and incomplete antibiotic use (53, 54). Community health workers (CHWs), embedded within their communities, play a pivotal role in sustaining these efforts by delivering personalised education on AMR, infection control, and hygiene practices, and guiding people in recognising early symptoms of infection and appropriate antibiotic use (55–57). Informal networks, including neighbourhood and household clubs, provide safe spaces for peer education, experience sharing, and guidance on appropriate antibiotic use. Complimentary visual materials on AMU and AMR, displayed in public places such as trading centres and health facilities, serve as consistent reminders that reinforce good practices (58). Periodic community assessments can help identify knowledge gaps, stimulate curiosity, and spark dialogue. When sustained over time, these efforts can integrate AMR awareness into everyday thinking and enable behavior change (59).

ii *Mass media and public broadcasts*

In the 21st century, the widespread availability of televisions, radios, and print media created powerful pathways for raising health awareness, particularly in rural and underserved areas where internet access is limited (60–63). Local media outlets can deliver standardized, evidence-based messages through talk shows, and music & drama programming, while public address systems in markets, public gatherings, residencies, and health facilities provide immediate, low-cost community engagement (64, 65). Print media, such as newspapers and information, education, and communication (IEC) materials, offer a lasting physical presence that supports repeated reference and reinforces self-awareness through clear, illustrated messaging in local languages (66). When strategically targeted and integrated into public awareness initiatives, these resources can expand reach, ensure consistent messaging, and strengthen self-awareness and engagement on AMR (52). However, major challenges persist, particularly limited funding to sustain mass media programming and the absence of curated, context-appropriate information that is accessible in local languages (67). Moreover, AMR and its associated concepts largely remain ‘Western’ constructs with no direct equivalents in most local languages in LMICs, making accurate translation and community-level communication difficult (68, 69). This linguistic and conceptual gap hinders effective public engagement and limits the localization of AMR awareness efforts in these settings (70).

iii *Digital tools and virtual engagement*

Recent technological advancements and expanded internet access (31, 32) have made digital platforms increasingly important for raising health awareness (71). Social media networks, including *TikTok, WhatsApp, Facebook, Reddit, YouTube, X* (formerly *Twitter*) and *LinkedIn*, are now central to how people, access and engage with health information. These platforms are well suited for distributing visually engaging, simplified content on topics such as symptom recognition, safe antimicrobial use, and where to seek help (33, 72, 73). Short videos, pictorial infographics, and gamified learning tools, designed in relatable formats, allow complex topics to be understood quickly. Peer-generated content and health influencer campaigns extend reach even further, especially when guided by HCPs or verified sources (74, 75). However, the proliferation of these digital platforms is accompanied by significant risks, including the rapid spread of medical misinformation, risks of privacy breaches, and the danger of users relying on unsafe algorithmic guidance or non-validated information (76, 77). Furthermore, a persistent digital divide, driven by disparities in gender, socio-economic status, and geography, risks exacerbating health inequities by isolating marginalized populations from these digital benefits (78). To ensure safety, digital self-care tools for AMR must be defined by rigorous validation and national certification against clinical accuracy standards, anchored within a clinical governance framework that aligns with national antimicrobial stewardship guidance. Such tools must also feature robust data protection safeguards and integrated escalation pathways, such as automated red-flag alerts, to guide users toward professional care when symptoms worsen, thereby ensuring that digital self-care remains a coordinated extension of the formal health system rather than an isolated practice.

Telehealth (Telemedicine) platforms, such as *Babylon Health, Mpharma, Ada Health, Rocket Health (Uganda)*, and *Doctor Anywhere*, enable real interaction with HCPs, strengthening awareness by providing timely information and guidance on emerging symptoms or infection control strategies outside the hospital settings (31, 32). They bridge critical gaps in the health system by offering convenient, on-demand access to HCPs, cutting travel-time and waiting periods, and bringing quality care to underserved communities (79). During the COVID-19 pandemic, telehealth proved indispensable ensuring continuity of care while minimizing exposure risks, enabling people to receive consultations, prescriptions, and follow-up services within communities (80, 81). These services facilitate early infection diagnosis and decision-making, guide individuals on AMU and infection control, and support diagnostic stewardship (82).

### 4.2 Self-testing and diagnosis

Self-diagnosis and self-testing are emerging self-care strategies, that enable individuals to identify health conditions and assess potential infections independently. Self-diagnosis involves recognizing health conditions based on symptoms, using available information from prior experience, books, digital platforms, or mobile health tools (83, 84). When supported by accurate tools, self-diagnosis can promote early identification of infections, informed decision-making, and appropriate AMU (85). Self-testing, in turn involves collecting samples such as body fluids and applying them to user-friendly devices, commonly lateral flow-based point of care (POC) rapid diagnostic test (RDT) kits, to detect indicators such as pathogen-specific antigens or antibodies (86), diagnosing infections early and facilitating timely decision-making (83). Self-testing and diagnosis reduce unnecessary clinic visits, lowering exposure to healthcare-associated infections, many of which are drug-resistant (87). In resource-limited settings, these practices ease the burden on overstretched health systems by allowing individuals to diagnose and manage simple conditions independently while reserving professional care for severe cases. During COVID-19 pandemic, self-testing and diagnosis allowed early diagnosis, allowing timely self-isolation, limiting transmission, and reducing unnecessary AMU (88).

Opportunities for expanding self-testing and diagnosis are growing, particularly with the increasing availability and accessibility of rapid POC test kits that can be applied for self-testing, empowering individuals to perform diagnostics at home or in community settings without requiring laboratory infrastructure. Successful examples include self-testing for malaria (89), HIV (90), and COVID-19 (88), where self-testing with RDTs has enabled early detection, reduced transmission, and supported informed self-management or linkage to care. Rapid POC tests for common viral infections that increase antibiotic misuse and overuse in LMICs, such as respiratory tract infections, include those for influenza (rapid influenza diagnostic tests), respiratory syncytial virus (RSV antigen tests), and COVID-19 (antigen or molecular POC tests), which help differentiate viral etiologies from bacterial ones to prevent unnecessary antibiotic use (91). Bacterial infections with rapid POC diagnostics include urinary tract infections (UTIs) using urine dipstick tests for nitrites, leukocytes, and other markers (92); streptococcal pharyngitis with rapid antigen detection tests for group A Streptococcus (93); and chlamydia with nucleic acid amplification tests (NAATs) adapted for POC use (94), these can enhance self-care by allowing quick, at-home or near-patient identification of bacterial etiologies, guiding appropriate antibiotic use or referral, reducing overuse, and promoting timely treatment adherence in community settings (95). The effectiveness of these interventions can be further enhanced by improving access to reliable health information and RDTs, and by integration with digital health tools, CHWs, and public education to guide interpretation and informed decision-making.

### 4.3 Self-treatment

Self-treatment and self-management are often used interchangeably, but they represent distinct concepts. While self-management is a broader approach that requires support from healthcare providers, self-treatment (sometimes referred to as self-medication) is narrower and involves independent use of over-the-counter (OTC) medications, traditional remedies, or other methods to manage health conditions without consulting a healthcare professional (96). While self-treatment can improve access to healthcare in resource-limited settings (97), it is also a major driver of antimicrobial misuse and overuse and AMR (98). Self-medication with antimicrobials remains highly prevalent in many LMICs, primarily driven by limited access to healthcare services, high consultation costs, widespread availability of OTC antibiotics, weak regulatory enforcement, and cultural beliefs favoring self-care (99).

Support groups play a vital role in improving treatment adherence, a key determinant of effective self-treatment, by providing a community-based platform for individuals to share experiences, challenges, and coping strategies. These include community-based adherence groups that meet physically or engage remotely to promote medication compliance, emotional support, and knowledge exchange. Evidence from HIV care shows that community adherence groups have improved retention in care and viral suppression among ART patients in Sub-Saharan Africa (100, 101). Similarly, peer-support groups for tuberculosis patients have been shown to enhance treatment completion rates and reduce defaulting (102). In chronic non-communicable diseases such as diabetes and hypertension, group-based education and peer-support initiatives have led to better self-monitoring, medication adherence, and lifestyle modification (103, 104). This approach can be effectively applied to enhance treatment adherence during self-treatment with antimicrobials through support groups that foster peer learning, accountability, and guidance on the appropriate use of antibiotics.

Strengthening self-treatment requires careful balancing of expanded access to timely care with the risk of inappropriate antimicrobial use and consequent acceleration of AMR. While self-treatment can improve accessibility in resource-limited settings where health system delays and access barriers are significant, it simultaneously introduces the potential for misuse, incorrect dosing, and unnecessary antibiotic consumption if not adequately structured (99). To mitigate this risk, any recommendations for self-treatment should be explicitly anchored in minimum safety requirements: use of quality-assured medicines within regulated supply chains; clearly defined clinical decision pathways and referral criteria to ensure timely escalation of care for non-resolving or severe illness; integration of feasible diagnostic support such as point-of-care testing where available; structured involvement of trained healthcare intermediaries, including pharmacists and community health workers, to provide oversight, counselling, and adherence support; and strong regulatory enforcement of over-the-counter antibiotic sales remains essential to prevent unregulated access (96).

#### 4.3.1 Self-treatment interventions for AMR containment

i *mHealth applications*

MobileHealth (mHealth) have become instrumental in providing users with educational resources on infectious conditions, updated treatment options, and optimal AMU. Applications (apps) such as *SteWARdS Antibiotic Defence* (105), *Skyscape* and *Medscape* (106), and *Firstline AMS App* (107), provide clear, user-friendly guidance on infectious disease management and optimal AMU, with offline functionality available on some apps for easy access in resource-limited settings.

ii *Wearable health devices*

Devices such as smartwatches, fitness trackers, and health-monitoring bands (e.g., *Apple Watch*, *Fitbit*, *Whoop Strap*, *Oura Ring*) provide real-time health data, such as vital signs, that aid in self-treatment and optimal AMU (108). These devices help with early infection detection, reducing unnecessary AMU, and supporting medication adherence (109). For instance, *Withings Thermo* monitors body temperature to help decide whether to continue over-the-counter treatments or seek antibiotic prescriptions (110). Wearables also promote preventive health behaviors such as those that track sleep, activity, and stress levels, which can boost immunity and reduce the need for antimicrobial interventions (111). Integration with telemedicine platforms, such as syncing *Fitbit* and *Garmin Vivosmart* with healthcare providers, allows remote monitoring and timely interventions, ensuring that antibiotics are only prescribed when necessary (112).

iii *Text messaging platforms*

Texting provides fast, direct communication at minimal cost, facilitating access to essential health information, medication reminders, and engagement with healthcare providers and peer support groups. In LMICs, SMS-based platforms can play a crucial role in infection self-treatment and AMS by providing structured guidance and reminders (113). As SMS do not require internet access (114), they are particularly valuable in areas with limited connectivity. In India, SMS reminders significantly improved adherence to tuberculosis treatment, reducing incomplete therapy and AMR (115, 116). SMS has been widely used in disease control, including outbreak response efforts (117). In Ghana, an SMS-based intervention successfully reduced the burden of malaria among children under five by enhancing awareness, promoting timely treatment, and improving adherence to preventive measures (118). Additionally, for over 10 years, SMS-based verification services have helped users in Africa and Asia confirm drug authenticity, reducing counterfeit medication use, preventing harm, enhancing clinical outcomes, and promoting AMS (119, 120).

Internet-based instant messaging platforms like *WhatsApp* have become widely used for health discussions in LMICs, where access to HCPs is constrained (121). WhatsApp offers a readily accessible, cost-effective platform for sharing vital health information, enabling remote consultations, and facilitating rapid communication, all of which are critical for effective self-treatment. Community-based WhatsApp groups enable individuals to share health information and experiences, seek informal medical advice, and discuss diseases and AMU. Group members can receive timely updates, disease outbreak alerts, counterfeit drug warnings, and IPC tips, all of which are essential for promoting optimal antimicrobial use. Many HCPs actively participate in these groups, providing an opportunity to offer basic guidance, correct misinformation, and promote appropriate AMU. In Kenya, WhatsApp-based initiatives enhanced community responsiveness and the efficiency of local emergency medical services in various ways (122), whereas in Sudan, WhatsApp improved the coping mechanisms of caregivers for children with neuro-disabilities (121), an approach that could be effectively applied to AMS initiatives. Automated messaging systems, powered by artificial intelligence (AI), are increasingly being integrated into self-care efforts. Chatbots like *ChatGPT* and virtual health assistants provide instant responses to users’ health queries and providing guidance on disease conditions, treatment, and optimal AMU (123). These tools can guide individuals on whether self-treatment is appropriate or if they should seek medical care (124).

iv *Social media*

Social media platforms have transformed the way people access health information and share experiences including self-treatment (33). Health organizations, public health campaigns, and even HCPs use these platforms to disseminate evidence-based information, such as AMR and AMS awareness during targeted campaigns such as WAAW, creating valuable opportunities for public education. TikTok, the short-form video platform, has become a powerful tool for engaging younger audiences through visually compelling content, making it an ideal medium for educating them about appropriate AMU (72). Platforms like *Reddit, Meta, and YouTube* also foster community-building, allowing individuals to seek advice from peers and share personal experiences related to self-treatment (125). While peer support can be valuable in some contexts, it also increases the risk of unsafe self-treatment practices, such as the inappropriate AMU (126). Health influencers who post educational and awareness videos on platforms like *Instagram, Snapchat, YouTube*, and *TikTok* hold significant sway over their followers (74, 75). These influencers reach a wide audience, particularly younger populations, offering a powerful platform to disseminate vital information for effective self-treatment, such as the appropriate AMU. However, it is crucial that their content is grounded in accurate, evidence-based guidance to prevent misinformation (127, 128).

v *Telehealth*

Telehealth offers convenient access to professional medical guidance, enabling informed self-treatment choices and reducing strain on healthcare systems (129). Platforms like *Teladoc* (130) and *Doctor on Demand* (81) allow patients to consult healthcare providers remotely, describing symptoms and receiving appropriate treatment recommendations, especially for mild infections. To address risks of misdiagnosis, patient mismanagement, and antimicrobial misuse, it’s essential to integrate evidence-based guidelines, clinical decision support tools, ongoing training for healthcare providers, symptom-checking algorithms, and patient education on proper antibiotic use into telemedicine initiatives (131). Safeguards for antimicrobial misuse such as clear prescribing protocols, and promotion of face-to-face consultations when needed are crucial (132). Telehealth enables effective symptom monitoring and follow-up care, allowing patients to report progress and address complications promptly (133).

### 4.4 Self-monitoring

Self-monitoring, the process by which individuals use digital health tools to track their health data, has become essential for proactive self-care (134). It enables real-time monitoring of symptoms, medication adherence, and physiological changes, supporting early infection detection and continuous health assessment, both of which are crucial for complementary self-care strategies such as self-treatment and self-diagnosis and testing (135, 136). By identifying potential health issues promptly, self-monitoring facilitates informed decision-making, promotes adherence to treatment, and help improve AMU (137).

#### 4.4.1 Self-monitoring interventions for AMR containment

Various tools such as digital medical devices and equipment, wearable devices, and mobile apps, enable early symptom detection, guide appropriate care-seeking, and support adherence to treatment regimens, all crucial for appropriate AMU (138–140).

Digital medical devices (e.g., thermometers, blood pressure monitors, and pulse oximeters) enhance real-time assessment of health status, while more advanced home-based tools (e.g., digital stethoscopes, otoscopes, and ECG monitors) can be integrated with self-treatment, self-testing and diagnosis, and remote consultation functionalities to enhance efficiency (141). Smart wearable devices (e.g., smartwatches and fitness trackers) continuously monitor physiological metrics (e.g., temperature, oxygen saturation, heart rate variability), generating data for early infection detection, monitoring treatment outcomes, and guide AMU (142–144). Additional innovations, including smart reminders, medication tracking apps and sensor-embedded packaging, reinforce treatment adherence and appropriate AMU (145). These technologies are especially beneficial in settings where they address healthcare access gaps while supporting appropriate AMU.

### 4.5 Self-management

Self-management refers to the ability of individuals to take informed, sustained action in managing their health and responding to illness in partnership with healthcare providers (146, 147). It involves the active participation of individuals in addressing their health conditions and maintaining well-being (148). This process depends on shared decision-making with healthcare providers to promote informed choices and better health outcomes. As a key component of self-care, self-management empowers patients and caregivers by fostering greater participation, self-determination, self-efficacy, and autonomy in addressing their health conditions, without compromising health outcomes (149). Self-management requires structured support from healthcare providers and the health system, including education, guidance, tools, and targeted interventions to enhance the capacity of patients and caregivers to manage health challenges effectively within their communities (148).

Self-management ensures that AMU decisions remain evidence-based and professionally guided. It enhances adherence to treatment regimens, reducing the risk of incomplete courses and subtherapeutic dosing (150). It empowers individuals to implement IPC practices, such as hand hygiene, wound care, and timely vaccination, limiting the spread of resistant pathogens (151–156). In community settings, where much of the antimicrobial use occurs without professional supervision (157), self-management helps individuals and caregivers make informed decisions about AMU and reduces dependence on unregulated medication practices (158).

#### 4.5.1 Self-management interventions for AMR containment

For self-management to effectively contribute to AMR containment, it must be embedded within a framework that ensures effective oversight by HCPs and the broader the health system (159). This includes standardized guidance, targeted patient and community education, and awareness initiatives that reinforce appropriate AMU and healthy behavior change (160).

Digital health tools can significantly enhance self-management and promote appropriate AMU (161, 162). Mobile Health apps installed on electronic devices enable symptom tracking, medication and health reminders, self-education, and direct communication with healthcare professionals, as seen with *M-TiBA* in Kenya (163) and similar platforms in South Africa (164). Telehealth platforms allow remote consultations, supporting timely decision-making while reducing unnecessary clinic visits (82). Wearable devices provide real-time monitoring of vital health metrics, guiding informed decision-making (165), while messaging platforms provide cost-effective channels for treatment reminders, health education, and instant engagement with healthcare professionals and peers (166, 167). SMS remains essential where internet access is limited (168, 169), while *WhatsApp* has become a widely used platform in Africa for personal and professional communication and has been successfully employed in resource-limited settings to improve AMU by delivering education content and supporting healthcare provider and peer engagement (73, 170). Social media, when carefully moderated, and integrated health campaigns and public health frameworks, can support dissemination of best practices and enhance remote professional support and supervision (170–172).

Directly observed therapy (DOT), a supervised approach designed to ensure medication adherence, plays a key role in self-management and can be implemented in several forms: facility-based DOT, where patients receive treatment at healthcare facilities (173); community-based DOT, conducted outside facilities such as in patients’ homes by trained workers (174); and video or tele-DOT, which enables remote observation by HCPs (175). Structured DOT has been highly effective in TB programs, improving treatment completion and cure rates (176). Given these successes, there is a growing need to develop similarly structured DOT models to support acute infection management in community settings, particularly for patients who cannot consent to admission. Such models could strengthen adherence and ensure appropriate AMU.

## 5 Challenges limiting self-care for AMR containment

While self-care offers a promising approach to improving AMU in communities, its implementation is constrained by multiple systemic, social, and behavioral barriers.

### 5.1 Information gaps and privacy concerns

Limited access to reliable essential information limits self-care strategies, restricting opportunities for individual behavioral change and the adoption of good AMU practices (51). Hard-to-reach communities, uneducated, and marginalised individuals lack the infrastructure, literacy, or institutional support to participate in AMR education efforts, limiting self-awareness (177). Misinformation, spread through social media networks and informal face-to-face interactions, continues to mislead the public by circulating inaccurate information that undermines understanding of AMR and mitigation measures (77, 178, 179). Common misconceptions and myths, such as “*antibiotics can treat all infections*” (180) or “*it’s no harm to stop taking antibiotics when you feel relieved”* (178), persist widely and remain major contributors to risk practices. Religious and cultural beliefs may also conflict with biomedical and public health approaches; while not inherently harmful, they can shape community and individual knowledge and awareness of AMR (181).

Effective self-care interventions require digital literacy, robust information technology (IT) infrastructure, and reliable digital connectivity (182–184). In resource-limited settings, many individuals lack access and the necessary skills to navigate digital tools and health platforms, hindering their ability to access vital health information and engage in self-care effectively (185, 186). This digital divide encompasses gender, socio-economic, geographical, and age-related disparities, significantly limiting adoption and effectiveness of self-care strategies in some communities. Marginalized groups, such as women, low-income populations, rural residents, and older adults, often experience unequal access to digital devices, internet connectivity, and technological skills, which exacerbates inequities in health information, digital health tools, and AMR awareness (78). This digital gap impacts infection detection and management in communities, leads to widespread antibiotic overuse and misuse, isolates individuals and communities from the health system, and reinforces the poor public awareness on AMR (187–189).

Self-care involves generation, transmission, analysis and interaction with individual health data. Weak regulatory oversight exacerbates concerns over privacy and personal health data, as health information is inherently sensitive and, without robust cybersecurity safeguards, remains vulnerable to breaches, unauthorized sharing, or misuse (190, 191). The growing reliance on digital tools and platforms for self-care, often involving the generation, storage, and transmission of health and demographic data amplify these risks (192). Breaches not only compromise individual confidentiality but can also erode public trust in self-care technologies, discouraging their uptake and limiting their potential in AMR containment (193). In LMICs, where cybersecurity regulations and enforcement mechanisms are underdeveloped, the risk is heightened by the use of third-party or non-health-specific digital tools that may lack compliance with health data protection standards (194).

### 5.2 Risks of misdiagnosis and mismanagement

Self-care carries inherent risks of misdiagnosis and misinterpretation of symptoms, delayed professional care, and self-medication, which can exacerbate inappropriate AMU (195–197). The proliferation of online tools and social media further increases the risk of misinformation, as untrained individuals may incorrectly interpret symptoms, leading to self-misdiagnosis (198–200). In addition, self-testing tools can produce false-positive and false-negative results, and counterfeit products remain a concern (201, 202). Self-testing and diagnosis cannot replace professional healthcare; trained healthcare professionals remain essential for accurate diagnosis and appropriate AMU (203). Integrating home-based testing and diagnosis with professional guidance can enhance effective diagnosis, case management, and appropriate AMU.

### 5.3 Risks of inappropriate antibiotic use

Self-care, while offering immediate relief for various illnesses, is frequently associated with self-medication and the often-associated inappropriate AMU, which drives AMR (204). The absence of professional oversight increases the risk of adverse drug reactions, harmful interactions, and delayed appropriate medical intervention, compromising both patient outcomes and AMR containment (196). Effective self-treatment depends on access to accurate health information and guidance, resulting in the over-reliance on digital health tools (205), yet many resource-limited settings face barriers such as limited infrastructure, internet, and data availability, leaving rural and impoverished communities excluded from these benefits (206, 207). In LMICs, these risks are amplified by cultural and behavioral factors, such as entrenched beliefs favouring self-medication, premature cessation of treatment once symptoms improve, and social pressures to share antibiotics, which undermine adherence and stewardship efforts (177). Poor water, sanitation, and hygiene (WASH) and IPC infrastructure and practices in communities, increases infection risks and the perceived need for antibiotics, perpetuating misuse (152). Weak governance and regulatory systems, including the weak policies governing community AMU, inadequate enforcement, and the circulation of substandard or falsified medicines, further facilitate inappropriate AMU, constraining the safe and effective implementation of self-care strategies in community settings (16).

### 5.4 Behavioral limitations

Effective self-care heavily depends on personal attributes such as motivation, discipline, and commitment, qualities that are often inconsistent over time (208–210). Activities such as symptom tracking, adhering to treatment plans, and following preventive measures are inherently boring, repetitive, and tedious, making it easy for individuals to lose interest or take shortcuts (211–214). This vulnerability is further compounded by negative traits like procrastination (215, 216), laziness (217, 218), or poor mental health (219, 220), which can significantly undermine adherence. On the other hand, overly motivated individuals may engage in over-diagnosis and over-correction (221, 222), leading to antimicrobial overuse and misuse (24, 223). Cultural beliefs and norms strongly shape personal health perceptions and decision-making, while practices that constrain autonomy, particularly among women and other vulnerable groups, can restrict timely self-care actions (224). Religious and cultural doctrines may further marginalize certain groups, such as gender and sexual minorities, reducing their access to information and services (224). Economic limitations, including poverty and financial insecurity, hinder prompt health-seeking behavior, consistent use of diagnostics, and completion of treatment courses (225).

## 6 Recommendations for enhancing self-care for AMR containment

Table 1 outlines multi-level strategies for strengthening self-care, requiring coordinated action across national programs, telehealth providers, and communities to contain AMR. National AMR programs in LMICs should prioritize integrating self-care into national AMR action plans, developing indicators to monitor self-care-related antimicrobial use, and standardizing community-facing AMR messages through unified national communication strategies. Crucially, these programs must regulate digital health tools and over-the-counter antibiotic access to ensure information accuracy and prevent the circulation of substandard medicines. To bridge the gap between policy and practice, there must be a concerted effort to strengthen community health worker support and train healthcare providers to guide local self-care initiatives. Meanwhile, telehealth providers should prioritize standardizing clinical protocols and embedding safety red-flag alerts in their workflows, while caregivers and self-carers must focus on informed decision-making, utilizing certified diagnostic kits, and adhering strictly to quality-assured treatment regimens.

**Table 1:** Multi-level strategies for strengthening self-care in AMR containment.

Strategies	National AMR programs	Self-care support programs	Telehealth providers	Caregivers	Self-carers
Policy integration	Integrate self-care strategies into NAPs-AMR integrate self-care indicators into AMR/AMU surveillance	Design and implement evidence-based self-care programs for AMU/IPC. Strengthen organizational and individual capacity to support long-term adoption.	Standardize telehealth protocols for self-care practices	Advocate for inclusion of caregiver roles in national AMR policies Engage with health authorities to ensure access self-care resources.	Advocate for equitable access to self-care tools. Engage with local health authorities to maintain access to reliable resources
Standardize health information and guidance	Disseminate standardized treatment guidelines and decision-support algorithms for common infections Develop a national-communication strategy for AMR	Design and implement targeted public awareness campaigns on AMR Implement public and community education programs to enhance self-care for AMR containment	Share evidence-based content with clients via digital platforms. Embed educational prompts and red-flag alerts in teleconsultation workflows.	Use trusted materials to guide symptom recognition and treatment adherence. Share AMR education resources within community networks.	Engage in self-directed learning on AMR and correct antibiotic use. Share educational materials and experiences with peer networks.
Strengthen capacity	Train HCPs and CHWs to support self-care for AMR Establish mentorship programs to support self-care for AMR	Provide technical assistance to key influencers (e.g., social media influencers) to combat misinformation. Integrate AI-based support into self-care tools	Train telehealth staff to deliver accurate AMR-related self-care guidance.	Strengthen skills in home-based infection control, AMU and recognizing when to escalate care. Support peers through informal coaching.	Develop skills in correct antibiotic storage, dosage, and course completion. Practice proper hygiene and preventive measures consistently.
Accessibility to quality antibiotics	Strengthen systems for equitable access to quality-assured antimicrobials. Enforce regulatory measures to limit substandard/counterfeit antimicrobials.	Implement programs to improve access in underserved settings (e.g., rural areas) Implement community-based AMS programs	Provide remote verification services for prescription validity. Guide clients to licensed suppliers	Verify antibiotic quality (e.g., packaging, expiry date). Encourage adherence to prescribed regimens	Purchase only from licensed pharmacies. Follow treatment guidelines and seek advice if uncertain.
Accessibility to self-testing kits	Ensure national procurement and distribution of validated self-testing kits for priority infections. Set quality control protocols for community-level supply chains.	Develop outreach programs for kit distribution in high-need areas. Train CHWs in community-level self-testing support.	Offer tele-support for correct use and interpretation of results. Track and flag abnormal patterns in user-reported data	Verify kit quality before use. Support proper sample collection and safe handling.	Use only certified kits. Follow instructions carefully and consult a professional when needed.
Availability of quality digital health tools	Approve and regulate certified self-care digital health tools for AMR containment. Ensure tools meet privacy, safety, and clinical accuracy standards	Distribute offline-capable self-care tools in low-connectivity areas. Include multilingual interfaces to improve reach.	Guide clients to quality tools. Enable real-time linkage to local services.	Learn and apply correct use of digital self-care tools. Share user experiences with providers for improvement.	Follow instructions for safe tool use. Report malfunctions or misinformation
Internet and digital connectivity	Expand infrastructure for digital self-care tools, prioritizing rural coverage. Negotiate zero-rated data use for AMR-related platforms with internet service providers (ISPs).	Deploy offline-capable self-care tools (e.g., SMS-based). Partner with ISPs for affordable access packages.	Offer hotline-based and SMS services as alternatives to internet. Provide asynchronous consultation options.	Use downloadable guides and offline apps where possible. Share local connectivity solutions with peers	Save/download AMR resources for offline reference. Offline consultations (peers and HCPs)
Strengthen regulatory measures	Enact robust data protection laws for digital self-care. Regulate online health information to curb misinformation.	Implement certification systems for self-care tools. Develop compliance checks for safe use.	Monitor and comply with relevant regulations. Remove unsafe or misleading content proactively	Advocate for caregiver rights in self-care contexts. Ensure informed consent in care activities.	Know your rights regarding data privacy and safe product access. Report unsafe tools or misleading advice.
Self-care financing	Allocate budget for self-care activities under NAP-AMR. Provide subsidies for priority self-care tools in vulnerable populations.	Analyze cost-effectiveness of interventions for scaling. Build sustainable funding partnerships.	Offer tiered-pricing for telehealth AMR services. Subsidize services for high-risk groups.	Budget for household self-care supplies. Access community support funds where available.	Plan personal health budgets to include self-care costs. Participate in pooled community purchasing schemes.
Research	Fund operational research on self-care’s impact on AMR. Evaluate integration into national health systems.	Study program effectiveness in changing AMU behaviours. Identify factors influencing community adoption.	Assess telehealth’s role in self-care for AMR containment. Pilot innovative remote diagnostics.	Document household-level enablers and barriers to self-care for AMR.	Participate in surveys on self-care practices. Provide feedback on tools used.

AMR, antimicrobial resistance; NAPs-AMR, national action plan for antimicrobial resistance; AMU, antimicrobial use; IPC, infection prevention and control; HCPs, healthcare practitioners; CHW, community health worker; AMS, antimicrobial stewardship. * The table presents evidence-informed recommendations synthesized from the narrative review and should not be interpreted as a summary of quantitative study findings.

Given the diversity of epidemiological, health system, governance, and resource contexts across LMICs, implementation of these recommendations should be context-specific. Rather than advocating a one-size-fits-all approach, these recommendations provide an evidence-informed framework that should be adapted to local priorities, capacities, and implementation challenges.

### 6.1 Policy development and implementation

Despite its growing importance, self-care continues to operate on the periphery of most national health policies and frameworks, often lacking formal recognition, dedicated funding, and structural integration into existing health systems. Most countries have yet to establish mechanisms to monitor or report on self-care interventions, and there are no standardized indicators for self-care within national health monitoring or AMR/AMU surveillance systems. This policy gap limits accountability, weakens coordination, and hampers efforts to assess the impact of self-care strategies on AMR containment. Moreover, fragmented implementation, poor regulatory oversight, and inadequate investment in community-based approaches hinder scalability and sustainability. These challenges highlight the urgent need to streamline and institutionalize self-care within broader health governance frameworks. Therefore, integrate self-care initiatives into national action plans for AMR containment and incorporate self-care indicators into AMR/AMU monitoring frameworks to ensure alignment with national priorities, enabling resource mobilization and coordinated action. National programs should institutionalize these indicators for routine monitoring to generate reliable data that can inform targeted interventions and track progress. Self-care support programs should integrate self-care into health systems’ strategies (226–229), and design evidence-based AMS and infection prevention interventions to strengthen community capacity for optimal AMU (230, 231). Telehealth providers should standardize protocols to align remote care with best practices and improve service quality, consistency, and accountability (232, 233). Caregivers should advocate for formal recognition of their role in AMU to secure the training, resources, and guidance needed to support appropriate AMU in households (49, 234), while self-carers advocate for equitable access to safe, effective tools and resources to inform decision-making (235–237).

### 6.2 Strengthen regulatory measures

Robust legal frameworks for data protection and regulation of health information are essential to safeguard user trust and promote engagement with self-care systems (238). National programs should enact and enforce these protections while regulating online health platforms to ensure accountability, mitigate misinformation, and prevent misuse of sensitive health data (239–241). Self-care programs should establish certification systems for self-care platforms and conduct regular compliance checks to guarantee information accuracy and reliability, and safety for users (242, 243). Telehealth providers should proactively remove unsafe content and strengthen data protection measures to protect client health information, reduce harm, and maintain service credibility (244, 245). Caregivers and self-carers should understand their rights, advocate for safety and privacy, and report misleading or harmful resources (246).

### 6.3 Standardize health information and guidance

Disseminate standardized treatment guidelines and decision-support systems for common infections to ensure consistency across all actors, improve self-care outcomes and AMU (247, 248). National programs should standardize information and communication on AMR to promote unified messaging and public understanding (36, 61). Self-care programs should implement targeted public awareness campaigns and community education on safe antibiotic use and infection prevention to enhance knowledge, promote responsible behaviors, and reduce infection rates (247, 248). To maximize reach and comprehension, AMR and self-care information should be made available in local languages and adapted to local cultural contexts. This will require collaboration with linguists and communication experts to ensure technical accuracy and cultural appropriateness. Translating and localizing health information is essential to bridge literacy gaps, enhance public trust, and enable individuals and communities, especially those in rural and underserved areas, to make informed decisions about antibiotic use and infection prevention and management. Telehealth providers should integrate prompts and red-flag alerts into their platforms to guide users and enable early recognition of complications and timely escalation of care (249, 250). Caregivers should actively seek and use trusted information, tools, and platforms to support accurate symptom recognition and treatment adherence in their communities (251, 252). Self-carers should engage in self-directed learning and share reliable educational resources with peers to strengthen individual and community knowledge and reinforce good AMU practices (253, 254).

### 6.4 Strengthen capacity for self-care

Strengthen the capacity of all actors involved in self-care through targeted measures to improve quality, safety, and effectiveness of interventions (35, 255). National programs should invest in training the public, healthcare providers, and CHWs to support self-care practices and optimize AMU, ensuring evidence-based guidance reaches self-carers and support systems (256, 257). Self-care programs should provide technical assistance to key influencers, including social media educators, to combat misinformation and promote accurate health messaging (258–260). Telehealth providers should equip staff to deliver standard AMU guidance and provide comprehensive client support, including follow-up and oversight, to improve adherence and early detection of complications (82). Caregivers should be supported develop skills in home-based infection control, appropriate AMU, and recognizing escalation triggers to prevent infections and ensure timely intervention (261, 262). Self-carers should build their capacity through continuous engagement with evidence-based information on appropriate antibiotic use (263).

### 6.5 Accessibility to quality antibiotics

Equitable access to quality-assured antimicrobials is essential for effective self-care and AMR containment. National AMR programs should strengthen antimicrobial regulatory and distribution systems to prevent substandard and falsified medicines (5). Self-care programs can implement community outreach in underserved areas and integrate antimicrobial monitoring into community supply chains to enhance access and appropriate use (264, 265). Telehealth institutions can verify prescription validity and direct clients to licensed suppliers, promoting access to safe antibiotics (266, 267). Caregivers and self-carers must check packaging, expiry dates, and ensure adherence to antibiotic treatment.

### 6.6 Accessibility to self-testing kits

National systems should procure and distribute validated self-testing kits for priority infections, supported by quality control measures to ensure accuracy, reliability, and safety (268). Self-care programs should expand access in high-need areas and train CHWs in proper kit use, increasing coverage, improving early detection, and supporting optimal AMU (89, 269, 270). Telehealth providers can provide remote guidance on test interpretation and connect users to professional care when needed, reducing diagnostic errors and facilitating appropriate AMU (271, 272). Caregivers should verify kit quality, assist with proper sample collection, and promote safe handling to ensure quality of results. Self-carers should follow instructions carefully and seek confirmation for positive or unclear results., enabling accurate diagnosis and appropriate use.

### 6.7 Availability of quality digital health tools

Regulation and certification of digital health tools for self-care by national programs are essential to ensure users have access to accurate, safe, and reliable platforms, thereby enhancing public trust and adoption. Self-care programs should support the distribution and use of offline-capable, multilingual tools for low internet-connectivity settings to expand reach and reduce health inequalities (273, 274). Telehealth providers guide clients to reliable tools and facilitate real-time links to local suppliers and services, ensuring timely access to quality tools and appropriate care (274, 275). Caregivers and self-carers should learn correct tool usage and report errors and misinformation (276, 277).

### 6.8 Internet and digital connectivity

Digital self-care depends on reliable digital connectivity, making offline access critical for rural and underserved areas to ensure equitable participation and timely care (278). National programs should expand internet and digital infrastructure and negotiate zero-rated data use specific AMR platforms service providers, enhancing accessibility and reducing barriers to effective self-care (279, 280). Self-care programs can deploy SMS-based tools and secure affordable data packages through strategic partnerships, enabling consistent engagement in low-connectivity settings (281). Telehealth providers should offer hotlines, asynchronous consultations, and other offline resources to maintain continuity of support and provide guidance when online access is limited (282). Caregivers and self-carers can use downloadable guides and SMS-based advice to access accurate information and make informed decision even without internet connectivity.

### 6.9 Self-care financing

Sustainable financing is essential to ensure long-term adoption and equitable access to self-care. National AMR programs should allocate budgets for self-care and subsidize essential tools for vulnerable populations, increasing affordability and access (5). Self-care programs can assess the cost-effectiveness of self-care interventions and develop strategic partnerships to secure long-term sustainability (283). Telehealth providers should implement tiered pricing and subsidies for high-risk groups., expanding access (284). Caregivers and self-carers should plan for self-care expenses and leverage community-based health financing initiatives to sustain self-care initiatives (285).

### 6.10 Self-care research

National programs should implement research on the impact of self-care on AMU and AMR containment and its integration into health systems to generate evidence for policy and programmatic decision-making (5). Self-care programs must evaluate intervention effectiveness and drivers of adoption to optimize strategies and improve community uptake (286). Telehealth providers should assess their role delivering remote care and pilot innovations such as AI-supported diagnostics, prescription, and monitoring to enhance effectiveness of self-care (287). Caregivers can document house-level barriers and enablers to inform design and implementation of self-care programs, while self-carers should participate in surveys and provide feedback to improve relevance, adoption, and outcomes of self-care initiatives.

## 7 Study limitations

This review is subject to the inherent limitations of a narrative, non-systematic design. Study selection was based on purposive judgment aligned with key conceptual domains rather than a comprehensive, reproducible search strategy, which may have introduced selection bias and limited the completeness of the evidence base. To improve the analytical rigor of the synthesis, the evidence for each self-care intervention was examined in terms of whether it was derived from directly from AMR containment literature, or from indirect evidence inferred from related health domains such as HIV. This distinction was applied during interpretation and was guided by considerations of biological plausibility and similarity of implementation mechanisms. Given the absence of a systematic search process and standardized risk-of-bias appraisal, the findings should be interpreted as a contextual interpretive synthesis rather than an exhaustive or definitive assessment of the available evidence.

## 8 Conclusion

Self-care represent a transformative approach to complement traditional health systems in combating AMR, particularly in resource-limited settings. By empowering individuals, service providers, and communities with accurate health information and digital tools self-care strategies can enhance early infection detection, reduce unnecessary healthcare visits, and promote appropriate AMU. However, equitable implementation requires addressing critical barriers such as digital divides, misinformation risks, regulatory gaps, and socioeconomic disparities through integrated policy frameworks, capacity strengthening, community engagement, research, and sustainable financing.
